# COUPLES LIVING WITH ADVANCED CANCER: RAMIFICATIONS OF SOCIAL ISOLATION AND BENEFITS OF AN EMBEDDED SOCIAL NETWORK

**DOI:** 10.1093/geroni/igz038.1964

**Published:** 2019-11-08

**Authors:** Victoria H Raveis, Sheindy Pretter, Monique Carrero-Tagle, Daniel G Karus, Avani Shah

**Affiliations:** 1 New York University, New York, New York, United States; 2 Touro College, New York, New York, United States; 3 University of Alabama, School of Social Work, Tuscaloosa, Alabama, United States

## Abstract

Cancer remains a leading cause of death, especially among older adults. While spouses are commonly involved in the provision of emotional and practical assistance to their ill spouse, their caregiving is not without cost. Although knowledge of an impending death permits preparation for the loss, a long and protracted illness, or one marked by intense caregiving demands, can deplete the well spouse’s personal resources, increasing the risk of morbid bereavement outcomes. Well spouses (n=138), aged 50 and older (mean age 63.6), 41% male, providing 8+ hours of caregiving to a spouse with advanced cancer and a life expectancy of 6 months or less were followed over the terminal illness period. Caregiving spouses’ anticipatory grief, depression and anxiety were all significantly, inversely correlated with sufficiency of social support, specifically tangible, informational and emotional support (p<.05). At 2-3 months post-death (widowed subsample, n=82), the surviving spouses’ grief was inversely correlated with informational support (p<.01) and their social re-engagement post-death was directly correlated with sufficient tangible support (p<.05). Their narrative accounts about the illness and post-death period reveal various demands and negative social encounters they experienced with family members and others during this period, as well as, documenting support received. Spousal death presents a significant adaptive challenge, particularly to older adults who must adjust to the dissolution of longstanding bonds and altered life circumstances. Awareness of these complex relationship issues, i.e. problematic network relationships, will enable clinicians to attend to the needs and preferences of bereaved spouses during this period of heightened vulnerability.

